# An offline correction method for uncompensated series resistance and capacitance artifacts from whole-cell patch clamp recordings of small cells

**DOI:** 10.1186/1471-2202-12-S1-P259

**Published:** 2011-07-18

**Authors:** Cengiz Günay, Astrid A Prinz

**Affiliations:** 1 Dept. Biology, Emory University, Atlanta, Georgia 30322, USA

## 

Whole-cell patch clamp recordings from small cells, such as in Drosophila, suffer from large series resistance (R_e) and capacitive artifacts [1,3,4]. While the series resistance error is caused by only the electrode, the capacitive artifact is caused by both the electrode and membrane capacitances. These artifacts cannot be compensated for with the standard amplifier circuits because of the feedback ringing and other instabilities caused by small cells.

Standard electrophysiological measurement procedures for determining ion channel parameters using the voltage-clamp protocol suffer the most from these artifacts because R_e affects the effective membrane voltage on the cell. For instance, a standard electrophysiological technique is to record multiple voltage protocols from multiple cells, average the traces from the same cells, and leak-subtract the average. This cell average trace is then normalized by the estimated capacitance of the cell and can be averaged across cells. During this procedure, an uncompensated R_e perturbs the expected outcome in two ways:

Within the same cell, R_e changes during the collection of multiple trials; usually increasing with time because of the cumulative clogging of the electrode tip.

Between cells, both electrode and seal leak parameters vary, which affect R_e and the leak that runs through it, both of which affect the membrane voltage, V_m.

We propose a simple and straightforward method and a publicly available Matlab implementation to address both of these problems. We use a standard model of electrode resistance and capacitance [2] together with an isopotential neuron model to simulate passive responses during recordings.

For each recording, we use voltage clamp steps that exhibit passive responses from the cell to fit the passive parameters of our passive model. This allows estimating passive parameters for each trial and independent subtraction of the artifacts. The success of this method is dependent on having access to non-leak subtracted recordings as otherwise R_e and its effects cannot be estimated. Using this method, we estimated errors from two different voltage clamp datasets. One surprising result was that variable R_e contributes more significantly than the variable capacitance, such that capacitance normalization fails to regularize responses. In summary, we propose this offline series resistance estimation and removal method to complement voltage-clamp experiments and to reduce errors during data analysis procedures.
